# A trigger model of apoptosis induced by tumor necrosis factor signaling

**DOI:** 10.1186/1752-0509-5-S1-S13

**Published:** 2011-06-20

**Authors:** Chang Gu, Junjie Zhang, Yingyu Chen, Jinzhi Lei

**Affiliations:** 1 School of Mathematical Sciences, Peking University, Beijing 100871, China; 2 The Key Laboratory for Cell Proliferation and Regulation Biology of Ministry of Education, Institute of Cell Biology, College of Life Sciences, Beijing Normal University, Beijing 100875, China; 3 Laboratory of Medical Immunology, School of Basic Medical Science, Health Science Center, Peking University, 38 Xueyuan Road, Beijing 100083, China; 4 Center for Human Disease Genomics, Peking University, 38 Xueyuan Road, Beijing 100083, China; 5 Zhou Pei-Yuan Center for Applied Mathematics, Tsinghua University, Beijing 100084, China

## Abstract

**Background:**

The ability of living cells to respond appropriately to apoptosis signals is crucial for the proper development and homeostasis of multicellular organisms. For example, viable cells must be stable enough to appropriately respond to apoptosis signaling so that an irreversible death program is only induced when apoptosis signaling reaches a certain threshold. Previous studies have introduced bistability models in which signaling by caspase-3 activity represents a key regulator of cell fate in response to apoptosis stimuli.

**Results:**

In this study, apoptosis induced by tumor necrosis factor (TNF) signaling is investigated, and a mathematical model without the requirement for bistability is proposed. In this model, rapid degradation of the active forms of caspases -8 and -3 are included, and TNF-signaling is found to induce a pulse of caspase-3 activation and trigger an irreversible death program. This result agrees with experimental observations. The ability of a cell to respond to, or resist, apoptosis stimuli is also discussed. Furthermore, the activation efficiencies of caspases -8 and -3 that are essential to a cell’s response to extracellular apoptosis stimuli are defined. Based on the simulations performed, it is observed that activation efficiencies must be sufficiently sensitive to appropriately compromise a cell’s resistance and effectiveness in response to apoptosis stimuli.

**Conclusions:**

Our results suggest that bistability may not be a necessary condition for the induction of apoptosis by TNF signaling. Rather, a sharp increase in caspase-3 activity might be sufficient to trigger the induction of an irreversible death program. Accordingly, regulation of caspase activity and degradation of active caspases is essential for a cell’s response to apoptosis stimuli.

## Background

Apoptosis is a genetically programmed cell death event that is crucial for development, tissue homeostasis, and the immune response of multicellular organisms [1]. Correspondingly, defects in apoptosis can result in a number of serious diseases including cancer, autoimmunity, and neurodegeneration. Cells exist in either a ‘survival’ state or are undergoing apoptosis, depending on their response to apoptosis [2]. In a ‘survival’ state, cells are stable and resistant towards low levels of apoptosis signaling. In contrast, cells undergo apoptosis that involves the initiation of an irreversible signaling pathway when apoptosis signals exceed a certain threshold. Nevertheless, the means by which cells determine their fate (i.e., survival or death) based on signaling activity is not well understood.

There are two pathways known to trigger apoptosis: an intracellular pathway that is initiated when the cell is severely damaged or stressed, and a signaling pathway that is induced when extracellular death ligands are bound by their cognate membrane-associated death receptors [3]. Furthermore, the central mediators of the apoptosis pathways includes a set of cysteine proteases that are part of a large protein family known as caspases. In recent years, several important caspases have been identified. In particular, caspase-8 has been identified as a key initiator of the death-receptor pathway, and caspase-3 is an executioner of apoptosis wherein different pathways converge. All known caspases possess an active site cysteine which can cleave the Asp-Xxx bonds of several target substrates. However, caspases are initially synthesized as enzymatically inert zymogens. To activate caspases, pro-caspases are proteolytically cleaved by upstream caspases (as is the case for caspase-3, -6, and -7), are activated by induced proximity (caspase-8), or are activated by holoenzyme formation (caspase-9) [3].

The tumor necrosis factors (TNF) family of proteins is a group of cytokines that can induce apoptosis [4]. Known TNF family members include TNF-*α*, TNF-*β*, Fas(CD95/Apo-1) ligand, and TRAIL (TNF-related apoptosis-inducing ligand), to name a few. TNF acts via the TNF receptor (TNF-R) to trigger apoptosis.

Furthermore, TNF-R is associated with pro-caspases through adapter proteins such as Fas-associated protein with death domains (FADD) and TNF-R-associated death domains (TRADD), resulting in the activation of caspase cascades that can irreversibly commit a cell to apoptosis [5]. Upon stimulation of TNF ligand, adapter proteins aggregate and form membrane-bound death signaling complexes. These death-inducing signaling complexes (DISCs) recruit and activate the pro-caspase-8 which undergoes proteolytic cleavage as part of this process [3]. Activated caspase-8 usually exists as a dimmer, and upon release from DISCs into the cytoplasm, activated caspase-8 is capable of inducing downstream signaling that results in the production of activated caspase-3 from cleavage of pro-caspase-3. For this process, there are two different pathways [6]. For example, in cells known as type I, a large amount of caspase-8 is released from DISCs which rapidly and directly cleaves caspase-3. In type II cells, activation of caspase-8 results in a signal that is amplified via a mitochondria pathway to induce activation of caspase-9, which then cleaves pro-caspase-3. The resulting activation of caspase-3 then triggers an irreversible cell death program in the nucleus [3,7], and this involve the inactivation of PARP and DNA-PK, two key enzymes involved in the homeostatic maintenance of genomic integrity [8,19].

Many efforts have been made to quantitatively study the process of cell fate determination in relation to the simulation of apoptosis [2,9-15]. In studies by Hua et al. [12], a computational model for the Fas-signaling pathway was developed (hereinafter referred to as the HCCSL model, also refer to reference [14] for a corresponding minimal model). This model integrated data currently available regarding signaling networks downstream of Fas activation, including both type I and type II pathways, up until activation of caspase-3. The main drawback of the HCCSL model is that degradations of activated caspases -8 and -3 are not considered. Consequently, the HCCSL model predicts that the caspase activities should be saturated after prolonged stimulation, even when Fas signaling is very weak. However, these results are not consistent with experimental observations where the activity of caspases -8 and -3 decrease after reaching a maximal value [10,16-18] (to be detailed below). In studies by Bentele et al. [10], a mathematical model was used to study CD95-induced apoptosis in type I cells. Using model predictions and experimental data, a threshold mechanism was identified in which apoptosis fails to be initiated if ligand concentrations fall below a critical level. Moreover, this threshold was highly sensitive to the concentration of c-FLIP, an inhibitor of caspase-8 activation and Fas-mediated apoptosis. However, the basis of this threshold remain unclear.

Bistability may be one reason for the existence of such a threshold [2]. Several models have been proposed to explain the bistability properties associated with apoptosis signaling pathways [2,9,13]. In these models, the co-existence of steady states involving the capacity for low or high levels of caspase-3 activity are presumed to be an “essential condition” [2]. Moreover, a positive feedback loop involving caspase-3 activation, with a role for inhibitors of apoptosis (IAP) proteins, is hypothesized to be essential for such a bistability [2,15]. Nevertheless, to the best of our knowledge, no experimental evidence directly supports this hypothesis. There have been experiments which show a peak of caspase-3 activity following exposure to apoptotic stimuli, and this level of caspase-3 activity is not the same as the steady state level. For example, Bentele et al. [10] demonstrated that simulation of the human B lymphoblastoid cell line SKW 6.4, with *5µg*/mol anti-APO-1, resulted in a rapid increase in concentration of cPARP, a reporter of active caspase-3. cPARP concentrations reached their maximal level after 20 min, after which they steadily decreased over 3 h. In contrast, when high concentration of ligand were used in parallel assays, no significant increases in caspase-3 activity were detected after 24 h of stimulation [10]. In mouse mammary MOD cells, p53-induced apoptosis resulted in the activation of caspase-3 after 6 h at 30°C, with activation of caspase-3 reaching maximal levels after 12 h [16]. At this latter timepoint, a drop in colony formation was also observed. Similar temporal kinetics were also observed for human Jurkat cells where levels of cleaved caspase-3 began to increase 2 h after the addition of anti-Fas, and then reached a maximum level by 4 h [17]. These results suggest that cell fate may be determined by a sudden peak in caspase-3 activity, referred to as a ‘pulse’, rather than its steady state levels. If this is the case, then bistability is not an “essential condition” for the apoptosis pathway.

Tawa et al. reported that the rapid degradation of active caspase-3 is dependent on the catalytic activity of caspase-3 [18]. Using a mathematical model, Stucki et al. further predicted that degradation of actived caspase-3 may be particularly relevant to long-living cells [15]. For the HCCSL model [12], the rapid clearance of actived caspase-3 after being induced by apoptosis signaling was not considered, but however, could be an important factor for the pulse increase in caspase-3 activity that has been detected.

This study investigates apoptosis induced by extracellular signaling using a mathematical model that excludes bistability as a prerequistite. The mathematical model used is based on the HCCSL model, yet accounts for the rapid degradation of activated caspases -8 and -3. Moreover, this study is intended to address one aspect of cell fate determination, particularly the predominant signaling mediated by caspase-8 as an initiator and caspase-3 as an executioner, instead of comprehensively modeling apoptosis signal pathways. Despite the restricted nature of this investigation, our model shows fair agreement with experimental results, both qualitatively and quantitatively. As a result, we are able to study the temporal behavior of caspase-3 activity upon stimulation of apoptosis, and explicitly define the activation efficiencies of caspases -8 and -3, which are essential for a cell’s response to extracellular apoptosis stimuli.

## Model and Methods

### Model structure and formulations

**Figure 1:**
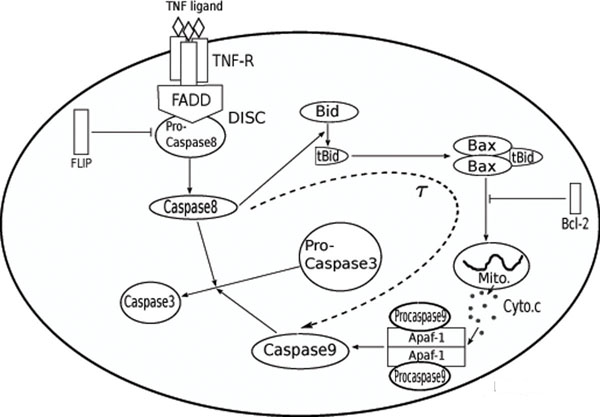
**A model for TNF signaling-induced apoptosis** An illustrated model of apoptosis induced by TNF signaling. TNF signaling induces the formation of membrane-bound death inducing signaling complexes (DISC), which subsequently recruit pro-caspase-8 and activate it by proteolytic cleavage. The active caspase-8 then cleaves various intracellular proteins, including pro-caspase-3, which results in caspase-3 activation and triggering of the cell death program. Caspase-8 cleavage of caspase-3 can occur through two separate pathways, either directly or indirectly, via mitochondria and caspase-9.

Figure 1 illustrates the apoptosis pathway being studied and the role for TNF-signaling. The main constituents were adapted from a Fas-signaling model previously established [12,14]. Upon binding of the TNF receptor (TNF-R) by TNF ligand (TNF), adapter proteins aggregate to form DISCs. These complexes then recruit and cleave pro-caspase-8 to generate active caspase-8, which in turn induces the cleavage of pro-caspase-3, either directly (in type I cells) or indirectly via mitochondria (in type II cells) [6]. In type I cells, a high concentration of caspase-8 is released from DISCs and directly cleaves pro-caspase-3 to activate caspase-3, which then triggers downstream signaling to the apoptosis pathway. Alternatively, in type II cells, caspase-8 signaling is amplified via mitochondria and pro-caspase-9 which results in the cleavage of pro-caspase-3. The latter amplification pathway includes the following main steps: activated caspase-8 enzymatically cleaves Bid to generate tBid. tBid is then associated with two Bax proteins (i.e., tBid:Bax2) and ‘activates’ mitochondria to release pro-apoptotic molecules such as cytochrome *c* and Smac/DIABLO. Once released, cytochrome *c* associates with Apaf-1 (Apaf) and pro-caspase-9 to form an apoptosome, the second initiator complex of apoptosis. The apoptosome generates active caspase-9 which then cleaves pro-caspase-3 to produce activated caspase-3.

In the following mathematical model, not every reaction was modeled. Instead, the time course of initiator protein concentrations (i.e., TNF receptor, caspase-8) and executor concentrations (i.e., caspase-3) of the apoptosis pathway will be focused on. Accordingly, there are five main components in our model, [TNF-R], [Casp8], [Casp3],  and [Casp3*], which represent the concentrations of bound TNF receptor, pro-caspase-8, pro-caspase-3, active caspase-8 and active caspase-3, respectively. The concentrations of both pro-caspases and active caspases are expected to exist in a steady state prior to the onset of the apoptosis signal. When TNF ligands are released and bind to their cognate receptors, downstream signaling is hypothesized to be affected by the stimulus, synthesis, degradation, and cleavage of pro-caspases. The time course of this process is modeled using the following set of differential equations(1)

Here, *V*’s represent molecule fluxes and are listed in Table 1. Detailed explanations of the fluxes involved are given below.

**Table 1:** Rate expressions and parameters

Rate	Rate expression	Parameters
Association of TNF to the receptor TNF-R,		*k*_on_ = 9.09 × 10^-5^ nM^-1^*s*^-1^
Dissociation of TNF,		*k*_off_= 1.00 × 10^-4^*s*^-1^
Activation of Casp8 through DISC,		*v*_1_ = 2.65 × 10^-3^*s*^-1^ *K*_1_ = *K*_2_ = 50nM
Activation of Casp3 directly,		*v*_2_ = 6.00 × 10^-5^nM^-1^*s*^-1^
Activation of Casp3 through mitochondria,		*v*_3_ = 2.60 × 10^-4^*s*^-1^ [Casp9]_0_ = 20nM *K*_3_ = 8nM *τ* = 30min
Synthesis rate		*v*_Casp8_ = 6.67 × 10^-4^nM*s*^-1^ *v*_Casp3_ = 4.00 × 10^-5^nM*s*^-1^
Degradation rate		*d*_casp8_ = 2.00 × 10^-5^*s*^-1^ *d*_casp3_ = 2.00 × 10^-7^*s*^-1^ *d*_Casp3*_ = 2.40 × 10^-4^*s*^-1^

In the above model, the equation for [TNF-R] is not essential for the apoptosis pathway, and is included only to reproduce the delay of caspase-8 activation that has been observed in experimental data (Fig. 3A). Furthermore, the coefficient 2 in the second equation is based on the dimerization of active caspase-8 that occurs.

**Figure 2:**
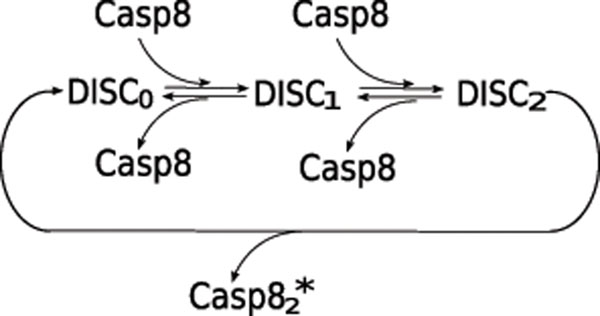
**A simplified model of caspase-8 activation via DISC** In this model, DISC*_i_* represents DISC with (*i*) number of pro-caspase-8 molecules.

The activation of caspase-8 is a complicated process that requires DISCs. For example, in the case of Fas-signaling induced apoptosis, these processes are composed of many reactions that include the recruitment of FADD, the binding of FADD to Fas receptor, the binding of FLIP or pro-caspase-8 to an adapter protein, and the generation of an intermediate cleavage product Casp8_2_: p41 [14]. Our model does not encompass the comprehensive scope of all of the reactions listed above. Instead, we have simplified these processes according to the state of DISCs, which is characterized by the number of pro-caspase-8 molecules present in each complex (Fig. 2). We omitted the states with three pro-caspase-8 molecules since DISCs can rapidly release activated caspase-8 dimers, thereby making the population of DISCs that are associated with three pro-caspase-8 molecules negligible. Furthermore, the reaction rate , as shown in Table 1, is obtained based on the assumption of quasi-equilibrium for the intermediate complexes DISC*_i_*. Here, the maximum number of DISCs is limited by the number of TNF receptors.

We assume a first order reaction whereby pro-caspase-3 is cleaved by caspase-8 in accordance with [14]. Similarly, the cleavage of pro-caspase-3 by caspase-9 is also considered to be a first order reaction. The activation of caspase-9 is usually induced by caspase-8 via mitochondria, and the detailed reactions of this pathway are omitted. They are replaced by a simplified assumption that caspase-9 activity depends on caspase-8 activity through a Michaelis-Menten type function. A delay, *τ*, is also introduced to represent the lag time needed to complete intermediate reactions mediated by the mitochondria. In the present model, *τ* = 30min for type II cells according to [6].

Molecule synthesis and degradation rates are assumed to be constants. Furthermore, the degradation rates of the active form of the caspases involved are assumed to be 2-3 orders larger than those of their inactive forms.

**Figure 3:**
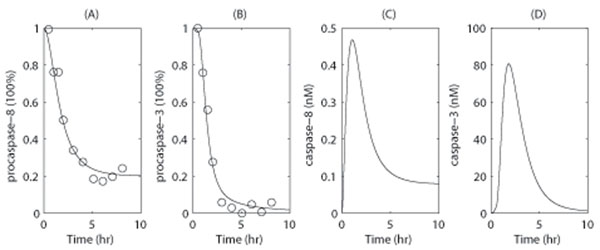
**Model simulation results for caspase activities** Model simulation results for pro-caspase-8 (A), pro-caspase-3 (B), caspase-8 (C) and caspase-3(D). In (A) and (B), the experimental data (circle dots) are retrieved from [12], Fig. 3, which include time courses for pro-caspase-8 and -3 in Jurkat cells in the presence of 100ng/ml FasL at room temperature. In the simulation, [TNF] was set to 2.0nM to mimic the stimuli associated with 100ng/ml FasL [12]. The total concentration of FasL receptor was also set to [TNF-R]_0_ = 10.0nM according to [12].

Parameters used in the present study are listed in Table 1, with most parameter values obtained from [14], and minor adjustments made due to the model modification and are summarized below. The synthesis rates, *v_X_*, activation rates, *v_i_*, degradation rates, *d_X_*, and coefficients *K_i_* are determined by fitting the model simulation to experimental data from human Jurkat cells treated with 100ng/mol FasL (Fig. 3). The delay, *τ*, based on [6], while other parameters were taken from [14]. The association and dissociation rates between ligand and receptor were based on [12], which describes Fas-signaling-induced apoptosis. These parameters could be modified to account for different types of ligands.

In our simulations, the initial conditions are assumed to be at steady state prior to the onset of stimuli (i.e., [TNF] = 0). Thus, [Casp8] = 33.33nM, [Casp3] = 200.00nM, and  as initial conditions [12].

In comparison with the original HCCSL model, two main simplifications have been applied in the current model. First, components of the reactions involved in the formation of DISCs were simplified, and are only represented by the relation between the output flux of activated caspase-8 dimers and input stimuli signals. The second simplification was to represent the amplification pathway mediated by mitochondria as the response of caspase-9 activity to caspase-8 dimer concentration, while having a delay to incorporated to represent the total time required by the intermediate steps. With these simplifications, we are able to reduce the original 35 variable equations to the current model of 5 independent values, and thereby reproduce the same dynamics as the HCCSL model (Fig. 3).

### Caspase-3 activity and cell apoptosis rate

Caspase-3 has been well-characterized as an executioner of apoptosis, by cleaving and inactivating PARP and DNA-PK, two enzymes that have been identified to have key roles in the homeostatic maintenance of genomic integrity [7,8,19]. Previous experiments have demonstrated that a pulse increase in caspase-3 activity is induced following exposure to apoptosis stimuli. For example, when the human B lymphoblastoid cell line, SKW6.4, was stimulated with *5µg*/mol of anti-APO-1, caspase-3 activity peaked, and then decreased [10]. In p53-induced apoptosis, irreversible apoptosis occurs even when caspase-3 activity is shown to be decreasing [20], yet the early stages of apoptosis are reversible [16].

In combination, the above observations suggest that the rate of cell apoptosis depends on temporal activation of caspase-3. Therefore, we propose the following formulation to represent the apoptosis rate(2)

Here, *r*_0_ is the basal apoptosis rate in the absence of executioner caspase-3, and *τ_d_* is the effective time of caspase-3. The Hill type function is used to represent the instant role of caspase-3. The basic assumptions for Eq. 2 are: (1) the downstream apoptosis signaling is triggered when caspase-3 activity exceeds a threshold, *K*_4_, and (2) the effect of caspase-3 is cumulative. Accordingly, it is straightforward to have the following equation for the number of surviving (*S*) and apoptotic (*A*) cells,

*dS/dt* = *r*_pro_*S — r*_apop_(*t*)*S*, *dA/dt* = *r*_apop_(*t*)*S*, *S*(0) = *S*_0_, *A*(0) = 0*.* (3)

Here *r*_pro_ is the proliferation rate (assumed to be a constant). The percentage of apoptotic cells is

*f_a_*(*t*) = *A*(*t*)/(*S*(*t*) + *A*(*t*)), (4)

and can be measured by flow cytometry. Therefore, the parameters in Eq. 3 can be determined by fitting *f_a_*(*t*) to experimental data.

### Caspase activation efficiency

Here we define the activation efficiencies for caspase-8 and -3, respectively, to measure their ability to generate active forms of caspase-8 and -3 in response to TNF stimuli.

First, we write down the variance equation when [TNF] = 0. Note that when [TNF] = 0, we have(5)

at steady state. Let *δX* to be the partial derivative (*∂X*/*∂*[TNF])|_[TNF]=0_. Performing partial derivative to Eq. 1 with respect to [TNF], and setting [TNF] = 0, we obtain following variance equation(6)

Here,  represents the total flux of caspase-3 activation. Setting the left hand side of Eq. 6 to zero, and solving for the steady state gives the following(7)

where *K*_0_ = *k*_on_/*k*_off_ represents the dissociation constant of the apoptosis ligand-receptor complex. The variances  and *δ*[Casp3*] given above measure the changes in levels of active caspases, and these are closely related to the resistance properties of a cell. Accordingly, the activation efficiencies of caspase-8 and -3 can be defined respectively as the coefficients in  and *δ*[Casp3*] of Eq. 7 as follows:(8)

and(9)

Then, using  and  in the present model:(10)(11)

Biologically, the activation efficiency *A*_Casp8_ measures the marginal effect the apoptosis ligand-receptor complex has on caspase-8 activation, and *A*_Casp3_ measures the marginal effect of caspase-8 on caspase-3 activation.

## Results and Discussion

### Caspase-3 activity exhibits pulse increase in response to apoptosis stimuli

The time courses of the concentration of pro-caspases and active caspases were monitored in our simulation for 10 h (Figure 3). A time-dependent reductions in levels of pro-caspase-8 and -3 were detected (Fig. 3, A&B), as well as the time course for caspase-8 and -3 activation (Fig. 3, C&D). In the latter case, both activated caspases exhibited a pulse increase in activity which reached a maximum level after approximately 2 h, and then decreased to low levels. These results agree qualitatively with previously reported experimental results [6,17,21], and this simulation reveals that the maximum level of activated caspase-8 occurs at low saturation (i.e., at ~ 2.8% of the maximum pro-caspase-8 concentration). We hypothesize that this is based on a reduction in DISC formation, as previously shown in type II cells [6]. In contrast, caspase-3 activity reaches its maximum at ~ 40% saturation approximately 30 min after caspase-8 reached its maximal level. At the very end of the simulation, caspase-3 activity reached a very low level due to caspase-3 degradation and a reduction in levels of pro-caspase-3.

**Figure 4:**
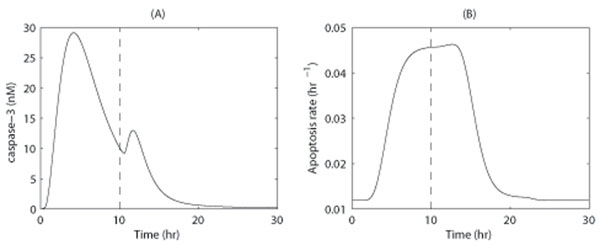
**Model simulation results for a model prediction** The model simulation results obtained for a cell is stimulated with a low apoptosis signal (i.e., 0.1nM FasL) for 10 h, followed by high apoptosis signal (i.e., 2.0nM FasL). (A) Time course of caspase-3 activity. (B). Time course of the apoptosis rate.

After exposure to apoptosis stimuli, pro-caspase-3 is cleaved to produce caspase-3, which is rapidly degraded. As result, pro-caspase-3 can be consumed even when stimuli are weak. In our model, a straightforward approach obtains the following prediction: If a cell is initially exposed to low levels of TNF signaling for a few hours, the pool of available pro-caspase-3 will be consumed. If high levels of TNF stimulation then subsequently occur, caspase-3 activity will not be as high as it would be in cells that were treated with high levels of TNF stimuli. The prediction is shown by Figure 4 with simulation time course of caspase-3 activity and rate of apoptosis in accordance with the treatment of a cell with 0.1 nM FasL for 10 h, followed by 2.0 nM FasL for 20 h.

### A plateau in the apoptosis rate of cells exposed to apoptosis stimuli

**Figure 5:**
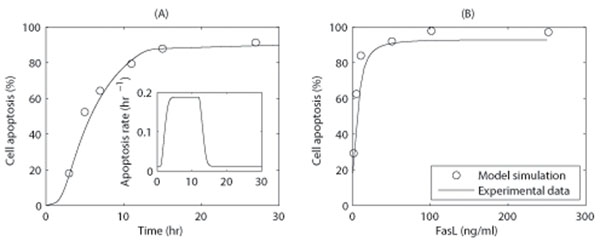
**Simulation results for cell apoptosis (*f_a_*(*t*))** (A) Time course for the cell apoptosis induced in Jurkat cells following exposure to 50ng/ml TRAIL. The control time point in the experiment corresponds to 3 h at the simulation. Experimental data were analyzed using a FACSCalibur flow cytometer (Figure S1 in the Supplemental Material). The inset shows the time course for the apoptosis rate. (B) Simulation results of cell apoptosis for Jurkat cells incubated with increasing concentrations of death stimuli 16 h. Experimental data were retrieved from [25], Fig. 5. The parameters used included: *r*_pro_ = 0, *r*_0_ = 3.31 × 10^-6^*s*^-1^, *τ_d_* = 11hr, *p* = 1.05 × 10^-8^*s*^-1^, *K*_4_ = 55.00nM, *n* = 4.0, and [TNF] = 1.0nM to mimic 50 ng/ml TRAIL. The proliferation rate was assumed to be zero based on the arrest of cell proliferation observed during the experimental period.

The apoptosis rate of cells following exposure to apoptosis stimuli is an important parameter in the study of cell’s response to apoptosis signaling. To determine the time dependence of a cell’s apoptosis rate, Jurkat cells were treated with 50ng/ml TARIL, and apoptosis was monitored. The experimental methods and results are shown by Figure S1  in Additional file 1 , and model simulations are shown in Figure 5.

In Figure 5, the apoptosis rates were obtained according to Eq. 2 and Eq. 3. TRAIL (Apo-2) is a member of the TNF family, and uses a similar mechanism as FasL to induce apoptosis [22-24]. Therefore, we used the same parameters listed in Table 1 to mimic the effect of TRAIL treatment, however, the parameters in Eq. 2 and Eq. 3 were adjusted to fit the observed apoptosis rates (Fig. 5A). From simulations, the cell apoptosis rate was observed to plateau after cells were exposed to apoptosis ligands (Fig. 5A, inset). In later stages, the cell apoptosis rate dropped to basal level due to a decrease in caspase-3 activity.

We also examined the response of Jurkat cells to different concentrations of TNF stimuli. Simulations show that Jurkat cells were observed to be sensitive to the stimuli, even when ligand concentrations were low (Fig. 5B). This appears to be in good agreement with previously reported experimental data [25].

### Caspase activation efficiencies are important for cell fate determination

Effectiveness and resistance are two important properties of an apoptosis response. To quantitatively analyze these properties, we defined the effective and resistance coefficients of a cell as follows, and demonstrate that they depend on the efficiency of caspase activation.

First, the average half-lifetime (*T*_1/2_) of cells is the time it takes for the proportion of surviving cells to be reduced by 50%. We assigned *T*_0_ to be the average half-lifetime of cells in the absence of an apoptosis stimulus. Correspondingly, the relative half-lifetime *T*_1/2_/*T*_0_ measures the decrease in cell life time that occurs in the presence of apoptosis signaling.

The effective coefficient (EC) measures the ability of a cell to trigger the death program when the apoptotic initiator signal is strong. Therefore, in the case of FasL-induced apoptosis, we define the EC as the relative half-lifetime that is exhibited in the presence of 100ng/ml FasL (i.e., [TNF] = 2.0nM in the model):

EC = (*T*_1/2_/*T*_0_)|_[TNF]=2.0_. (12)

For this definition, a smaller EC represents a more effective response to apoptosis signals.

The resistance coefficient (RC) measures the ability of a cell to resist minor apoptosis signals, and is defined as the change in the relative half-life time when there are minor apoptosis signals as follow:

RC = -(*∂T*_1/2_/*∂*[TNF])/*T*_0_|_[TNF]=0_. (13)

The negative sign ensures that a positive resistance coefficient will be obtained, and a smaller RC value represents better resistance.

It is important for a cell to simultaneously exhibit good resistance and effectiveness. However, this is not always possible since good effectiveness (i.e., a low EC value) usually implies bad resistance (i.e., a high RC value) (see Figure S2 in Additional file S2 in the Supplemental Materials for simulation results).

**Figure 6:**
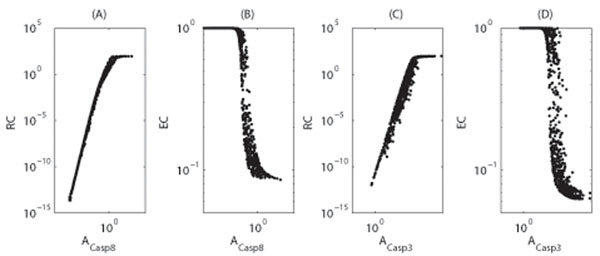
**Dependence of resistance and effective coefficients on the activation efficiencies** Dependence of resistance and effective coefficients on the activation efficiencies of caspase-8 (A-B) and caspase-3 (C-D). (A-B) The parameters were chosen from 10^-5^ <*v*_1_,  and 10 <*K*_1_(= *K*_2_) < 1000, and other parameters were taken from Table 1. For each set of parameters, the model equations were solved to obtain the resistance and effective coefficients, which are represented by one point in the figure. (C-D) The parameters were chosen from 10^-5^ <*v*_2_, *v*_3_, *d*_casp3*_ < 10^-2^ and 1 <*K*_3_ < 100, and other parameters were taken from Table 1.

To gain insight into how different coefficients in our model affect a cell’s response, the dependencies of RC and EC on activation efficiencies were investigated as shown in Figure 6. These results show that as activation efficiencies increases, so does resistance, yet effectiveness decreases. These results suggest that the activation efficiencies need to be well-adjusted in order to ensure a cell’s proper response to apoptosis signals, i.e., small values for both RC and EC.

In the present model, according to Eq. 10-11, activation efficiencies are determined by degradation rates and the coefficients *K_i_*(*i* = 1, 2, 3). Biologically, these parameters are associated with a family of genes that encode potent caspase inhibitors known as inhibitor-of-apoptosis (IAP) proteins [13,26-29]. For instance, the parameters *K*_1_ and *K*_2_ relate to the inhibition of apoptosis by cFLIP which directly inhibits the activation of caspase-8 by competing with pro-caspase-8 for binding to FADD [6,14,30,31]. In additional, *K*_3_ represents the modulation of caspase-3 activation that is mediated by a mitochondrial pathway involving Bcl-2 family members [3,6,12]. Correspondingly, overexpression of cFILP or Bcl-2 have been shown to block CD95-induced apoptosis [6]. However, further studies are needed to clarify the effect of these parameters on the cellular response to apoptosis signals.

## Conclusions

In this paper, a simplified mathematical model of TNF-induced apoptosis was developed based on the Fas-signaling pathway proposed by Hua et al. [12] and Okazaki et al. [14]. Our model exclude most of the detailed reactions involved, include rapid degradation of caspase-8 and caspase-3, and was focusing on the activation of these two caspases in response to TNF stimuli. Rapid degradation of caspase-3 has previously been reported [18], but this was not considered in the model by Hua et al. [12]. According to our model, a cell shown a pulse increase in caspase activations after exposure to apoptosis stimuli, and this was because of the rapid degradation of these caspases after being induced. This result is in contrast with the properties of bistable steady states which have been suggested by other models [2,9,13]. However, our results agree qualitatively with experimental data [6,17,21]. As such, our model indicates that a pulse increase in caspase-3 activity is sufficient to trigger the irreversible apoptosis response. Moreover, the dynamic properties of these cell signaling pathways suggest that the timing of cell signaling is also an important aspect of inducing apoptosis. Correspondingly, the timing of cell signaling has been implicated in experiments of p53-induced apoptosis [16,20]. Overall, a phenomenological cumulative relationship between the rate of cellular apoptosis and caspase-3 activation is proposed, and is in good agreement with experiment data.

In this paper, we also defined the EC and RC of a cell in order to facilitate the quantitative analysis of a cell’s response to apoptosis signals. In this study, caspase activations was found to be essential for the effectiveness and resistance of a cell. In addition, we proposed quantitative definitions to represent the activation efficiencies of caspase-8 and caspase-3 in terms of protein degradation rates and model parameters that are related to the activation of apoptosis inhibitors. Our results suggest that caspase activation efficiencies can be regulated by inhibitors such as X-linked inhibitor of apoptosis protein (XIAP), cFLIP and Bcl-2 family members, which have been shown to be essential for the apoptosis pathway. Additional studies will be needed to further investigate the regulation of apoptosis inhibitors in the apoptosis pathway.

## Competing interests

The authors declare that they have no competing interests.

## Authors contributions

JL designed the studies, analyzed the simulation data, and wrote the paper. CG and JZ developed the model. CG performed the numerical simulations. YC performed the experiment. All authors have read and approved the final manuscript.

## Supplementary Material

Additional file 1 Experimental result of Jurkat cells apoptosis induced with TRAIL. Click here for file

Additional file 2 Simulation results of the dependence of resistance and effective coefficients to the activation efficiencies of caspase-8 and caspase-3. Click here for file
