# Supplementary material for: A trigger model of apoptosis induced by tumor necrosis factor signaling
Source: BMC Syst Biol. 2011 Jun 20;5(Suppl 1):S13. doi: 10.1186/1752-0509-5-S1-S13 (PMC3121113; doi:10.1186/1752-0509-5-S1-S13)
Supplement: Additional file 1 — Experimental result of Jurkat cells apoptosis induced with TRAIL. [file 1752-0509-5-S1-S13-S1.pdf]

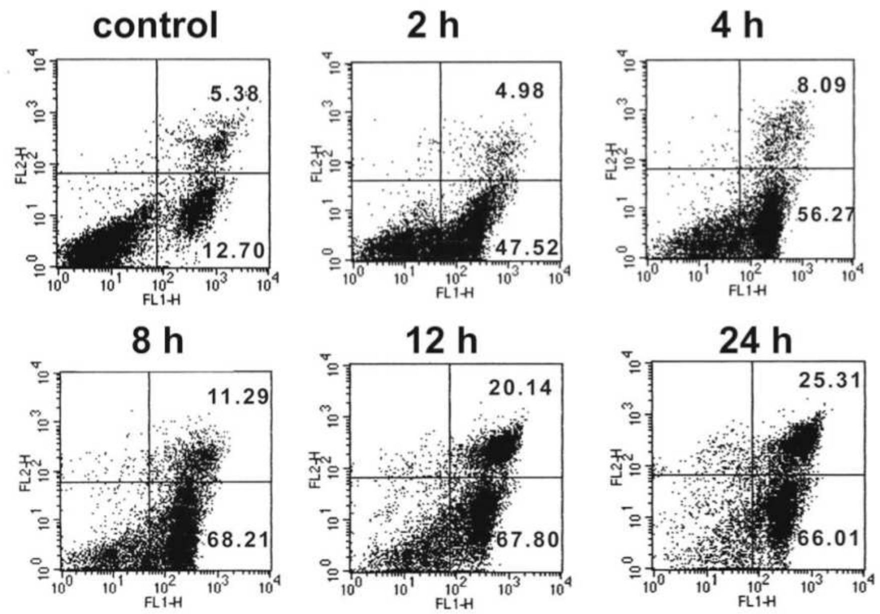

Figure S1. Jurkat cells apoptosis induced with TRAIL (50ng/ml) in a time dependent manner. Jurkat cells were treated with TRAIL for different time (0, 2, 4, 8, 12, 24 h), then washed twice with PBS, and resuspended in 200 μl binding buffer (10mM HEPES, pH 7.4, 140mM NaCl, 1mM $MgCl_2$, 5mM KCl, 2.5mM $CaCl_2$). FITC-conjugated Annexin V was added to a final concentration of 0.5 μg/ml. After incubation for 20 minutes at room temperature in the dark, PI was added at 1 μg/ml, and the samples were immediately analyzed on a FACSCalibur flow cytometer (Becton Dickinson, USA).
