# Supplementary material for: A trigger model of apoptosis induced by tumor necrosis factor signaling
Source: BMC Syst Biol. 2011 Jun 20;5(Suppl 1):S13. doi: 10.1186/1752-0509-5-S1-S13 (PMC3121113; doi:10.1186/1752-0509-5-S1-S13)
Supplement: Additional file 2 — Simulation results of the dependence of resistance and effective coefficients to the activation efficiencies of caspase-8 and caspase-3. [file 1752-0509-5-S1-S13-S2.pdf]

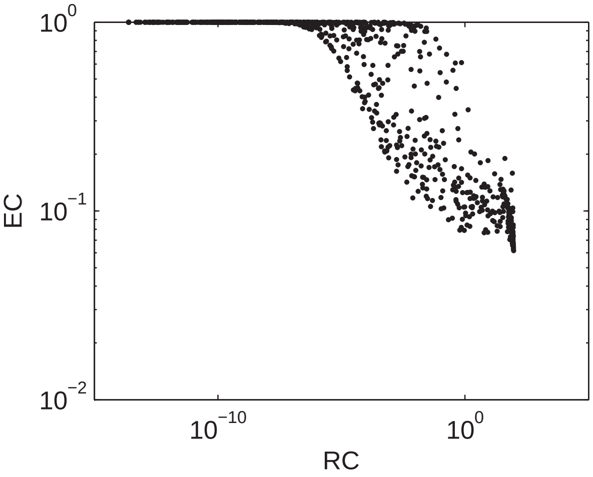

Figure S2. Simulation results of the dependence of resistance and effective coefficients to the activation efficiencies of caspase-8 (A-B) and caspase-3 (C-D). In (A-B), the parameters are chosen randomly covering the range $10^{-5} < v_1, d_{\mathrm{Casp8}_2^*} < 10^{-2}$ and $10 < K_1(= K_2) < 1000$, with other parameters taken from Table 1. For each set of parameters, the model equations are solved to obtain the resistance and effective coefficients, and to give rise to one point in the figure. In (C-D), the parameters are taken from $10^{-5} < v_2, v_3, d_{\mathrm{Casp3}^*} < 10^{-2}$ and $1 < K_3 < 100$, and other parameters from Table 1.
